# The Woman’s Department

**Published:** 1912-12

**Authors:** 


The average parents treat their children as if they were imbeciles and idiots.

Now, reader, don't get angry, but kindly wait and I will prove my statement:

You tell your child not to do something. He will say, "Why not, mother?"

What answer do you make? Oftener than otherwise, I have heard mothers and fathers say, "Children should do as they are told, and not ask questions," or, "You must not do it, simply because I don't want you to."

Is that fair and just to your child? If he were as large as you are, would you give him such an answer? Who else but you is there to explain the whys and wherefores of this new and strange life to him?

If only we *would* remember that the mind of a child is a vast, idealess plain that we are to cultivate, and that the concrete is more interesting to a child because he has no ideas for comparison, we would get much better results.

You tell your child to brush his teeth. He complains that the tooth brush scratches. Why not tell him that his teeth are his pearls, and that they will be beautiful and lasting only if he cares for them? Show the child the garbage can, and tell him that his little stomach would get in such condition if he allowed the food to decay between his teeth and drop into his stomach. He would become intelligently particular about the cleansing of his mouth after meals.

Did you ever explain to your child as you bathed him that his body is a temple where God lives; that he must keep the outside clean by bathing, and that the mind must be kept pure and sweet by good, kind deeds and thoughts? He will be interested immediately if you tell him that his body is like a church, and call his attention to the fact that churches should always be clean and beautiful. Explain to him that a good man or woman who is strong and robust is a much more beautiful temple than a bad, vicious, sick man or woman. Just try this and see if your baby's eyes do not twinkle with the light of intelligent understanding.

Your child asks where he came from. Do you give him a sane answer? An idiot or imbecile would be satisfied with the bare statement that the stork brought him. A bright, intelligent child is not, and so he keeps asking where babies come from—and after a while he finds some one who tells him. Too often this story is told him by some evil-minded playmate. He decides that there must be something wonderfully mysterious about these facts, and where there is mystery there is always curiosity. He keeps asking questions and making observations, until he reaches a few conclusions, one of which is that you—*his own mother*—have not told him the truth. He often decides that there must be something unclean about the matter, and that for this reason you have refrained from talking to him about it.

What might have been made holy and sacred to him becomes unmentionable and obscene.

What has the mother lost in this transaction?

She has lost an opportunity to make sacred and holy the divinity of sex; she has lost his *complete* confidence; and, while she may, in a measure, restore it, mend it, if you please, there remains the inevitable fate of the broken china—it can never be just the same again. And, last but not least, she has lost the most glorious privilege that motherhood brings—the sealing of that bond of understanding and mutual confidence that makes her child her very own in spirit and in truth.

Why motherhood, with the sufferings and pangs of birth, if not for the joy of looking into the depths of the child's eyes and telling him that he is bone of your bone and flesh of your flesh?

JOSEPHINE DANIEL.

---

We note with delight the fact that the clubwomen of the State are discussing and advocating the teaching of moral prophylaxis and social hygiene in the home and school. At the recent meeting of the Texas Federation of Women's Clubs, Dr. Mary Burnham of San Antonio delivered a very forceful address upon "Social Ethics." "The solution of the problem," she said, after picturing conditions as she knew them after sixteen years' experience as a practicing physician, "lies with the women of Texas." She urged the guidance of the young in such ways that they will reach maturity with an intelligent understanding of those things of which, through parental diffidence or neglect, so many learn in perverted and harmful ways. "This is a man-moral world now," she said in characterizing present conditions. "The education of the women," she gave as its solution, urging every club member to go home from this convention and give a determined and intelligent study to subjects of sex and hygiene and the taking up of the subject consistently with the girls and boys in the schools and colleges of the State.

---

THE State that requires a man to study two years before a license as druggist is given him; that makes a young lawyer or doctor study three years before being permitted to practice, ought to require the young man or woman to pass an equally rigid examination before license is given to found an American home and set up an American family.—Newell Dwight Hillis, D. D.

